# Disentangling interoception and its links to cognitive functioning in fibromyalgia

**DOI:** 10.1038/s41598-025-15087-5

**Published:** 2025-08-26

**Authors:** Mariana Agostinho, Manuel Luís Capelas, Fernando Manuel Pimentel-Santos, Rita Canaipa

**Affiliations:** 1 Center for Interdisciplinary Research in Health (CIIS), Universidade Católica Portuguesa, Lisboa, Portugal; 2 Faculty of Health Sciences and Nursing, Universidade Católica Portuguesa, Lisboa, Portugal; 3 The Cheryl Spencer Department of Nursing, Faculty of Social Welfare and Health Sciences, University of Haifa, Haifa, Israel; 4 NOVA Medical School, Universidade Nova de Lisboa, Lisboa, Portugal; 5 Hospital de Egas Moniz, ULS de Lisboa Ocidental, Lisboa, Portugal

**Keywords:** Fibromyalgia, Interoception, Executive functioning, Memory, Neuroscience, Psychology, Rheumatology

## Abstract

Patients with fibromyalgia experience pain, cognitive dysfunction, and atypical interoception. However, it is still unclear whether the changes in interoceptive processes have consequences in managing cognitive tasks. The current study investigates the relationship between interoception and cognition in fibromyalgia. Twenty-nine fibromyalgia patients completed clinical questionnaires, the Digit-span, and the Stroop test. Interoceptive Accuracy (IAc) was measured by the heartbeat detection task, Interoceptive Awareness (IAw) via confidence ratings, and Interoceptive Sensibility (IS) via the Multidimensional Assessment of Interoceptive Awareness questionnaire. IAc was found to be positively associated with Digit-span forward (p = 0.008), total (p = 0.004), and Stroop scores (all p ≤ 0.045). IS correlated negatively with Digit-span backward (p ≤ 0. ≤ 0.022) while IAw was negatively associated with Digit-span forward (p ≤ 0.034), total (p ≤ 0.008), and Stroop scores (all p ≤ 0.038). Moderation analysis revealed that IAc predicts Digit-span backward as symptom severity increases (p = 0.045). Despite the absence of a control group, this study provides evidence of the detailed relationships between interoception and cognitive abilities in fibromyalgia. While detecting and efficiently using body signals may be an essential tool for self-regulation in managing cognitive tasks, a higher ability to regulate body signals may exhaust cognition and predispose the patients to lower cognitive performance.

## Introduction

Interoception, the internal sense of bodily state, is the ability to detect, represent, and interpret various body signals, such as heartbeat, thirst, taste, itch, or pain. It is essential to orchestrate conscious and unconscious homeostatic (or allostatic) adaptations, drive emotional and cognitive responses, and contribute to behavior selection^1,2^. The study of interoception has proven difficult, and the debate concerning interoceptive dimensions is ongoing, reflecting the concept’s multidimensional nature and the challenges of measuring subjective concepts^3–5^. One of the most accepted perspectives considers three main components of interoception^3^: (1) Interoceptive accuracy (IAc), the ability to detect body signals in interoceptive tasks, measured, for example, by the heartbeat detection task^6,7^ where the number of heartbeats reported by the individual is compared to the actual number of heartbeats recorded in an electrocardiogram; (2) Interoceptive sensibility (IS), the self-reported ability to perceive and regulate body signals assessed by questionnaires such as Multidimensional Assessment of Interoceptive Awareness (MAIA)^8^; Finally, (3) Interoceptive awareness (IAw) is the relation between the individual’s confidence in detecting his heartbeat and the actual accuracy.

Enhanced interoceptive abilities allow early detection of bodily harm signals, self-regulation, and recovery behaviors that promote health and healing^9–11^ (for a review see Brewer et al.^12^). The development of interoceptive abilities may be essential for individuals facing chronic pain conditions where the perception of body signals must be integrated to pursue appropriate health behaviors and self-regulation strategies^13^. However, data on chronic pain conditions describe atypical IAc^14–19^ and Interoceptive Sensibility^20^. Specifically, in fibromyalgia, a chronic pain condition with pain, fatigue, sleep problems, and cognitive dysfunctionas core symptoms^21–23^, research on IAc has shown mixed results^15,24–27^. A few studies suggest lower IAc in comparison with non-clinical individuals^15,27^, but others failed to corroborate these findings^24–26^. On the IS dimension, evidence supports the view of enhanced abilities in fibromyalgia patients and other chronic pain patients than in controls^16^. Another question that remains open is the relationship between interoception and other fibromyalgia symptoms beyond emotional and pain symptoms, such as cognitive dysfunction. Changes in cognitive abilities are a hallmark of fibromyalgia syndrome^28,29^ and one of the most debilitating characteristics of this condition. Studies on healthy individuals supported the notion that Interoception is related to cognitive abilities^30–33^: disrupting interoceptive inputs by increasing cytokine activity due to vaccination impacts the activity of the right insula while performing a cognitive task^30^, lower interoceptive abilities are related to lower memory performance^31^ and lower performance on emotional Stroop^32^. Patients with temporal lobe damage show difficulties in interoceptive functioning that are related to their memory deficits^33^. These findings highlight the importance of further investigating how interoception is related to cognitive functioning in conditions that show altered perception of body signals and cognitive dysfunction, as well as the urge to explore new therapeutic approaches.

The current study investigates the relationships between interoceptive dimensions (accuracy, sensibility, and awareness) and executive functioning performance (working memory and inhibitory control) in patients with fibromyalgia. Given that interoception is required to regulate internal states in the face of cognitive and emotional challenges, we hypothesize that fibromyalgia patients who demonstrate difficulties in the ability to listen to their internal states will also be impaired in the ability to guide and adjust their behavior according to the demands of the cognitive tasks.

## Methods

### Participants

Thirty fibromyalgia female patients were recruited through physician referrals from the Rheumatology department of a local hospital in Lisbon. The physician described the study’s general aim (to study the ability to report pain and other subjective measures) as well as the procedures. Those willing to participate provided their contact information. Only those with fomal diagnosis from a physician, according to the 1990 American College of Rheumatology (ACR) criteria^34^ and the recent^35^ diagnostic criteria, were invited to participate. Additional inclusion criteria were age above 18 years old, female, on stable medication therapy for at least 4 weeks prior to their participation and provide signed informed consent. Exclusion criteria included pregnancy or breastfeeding, persistent or severe infection within the previous 30 days, diagnosed psychiatric conditions, comorbidity with other rheumatic disease, uncontrolled medical condition (e.g., diabetes mellitus, ischemic heart disease) or signs of demyelinating disease.

A power analysis using the G*Power Software [^36^, version 3.1.9.6] was conducted to ensure sufficient statistical power to detect correlations between interoceptive main outcomes and cognitive tasks and based on an effect size of 0.5 (correlation coefficient |p|), a power of 0.8, a significance level of 0.05 indicated that the sample size needed to meet the desired statistical power was 26.

As an exploration analysis, we also conducted a power analysis to detect the moderating role of clinical symptoms such as FIQ Symptoms and to assess the increment of explained variance due to the interaction between independent and moderator. This analysis was based on a multiple regression and an R2 increase for a statistical power of 0.8 and a large effect size of 0.30 with an alpha set at 0.05. This power analysis revealed that recruiting 29 participants would provide sufficient power.

### Interoceptive assessment

#### Interoception accuracy (IAc)

The heartbeat detection task (HDT), developed by^6^, measures IAc, comparing participants’ self-reported heartbeats counted to objective heartbeats collected via an electrocardiography (ECG). Participants were seated in a quiet room free from distractions, in a comfortable chair with arm support. They were instructed to remain relaxed, avoid sudden movements, and focus on their inner experiences without checking their pulse or using any aids. Ag/AgCl electrodes were attached to them according to Eithovens’ triangle, connected to a BITalino (BITalino, PLUX – Wireless Biosignals) device for ECG recording.

The task began with 5-min resting period followed by a training trial of 15 s. Then, participants completed three counting phases of different durations in a fixed order (25, 35, 45 s) with 30 s of rest in between trials. Each counting phase began and ended with a tone, during which participants were instructed to silently count heartbeats based on bodily sensations without physical or breathing manipulations, reporting counted heartbeats at the end of each phase. The Interoceptive Accuracy outcome derived from the HDT was calculated:

$$\frac{1}{3}\sum 1-\frac{\left|recorded heartbeats-reported heartbeats\right|}{recorded heartbeats}$$

#### Interoception awareness and insight

After each trial of the HDT, participants rated their confidence in their ability to accurately count the heartbeats on a scale from 0 (“not confident at all”) to 10 (“extremely confident”). Three main outcomes were calculated: confidence average (CA), within-subjects correlation (correlation), and interoception discrepancy index (DI). CA was calculated by averaging confidence ratings of all the trials. The correlation outcome was calculated using a within-participant correlation between the IAc outcomes and the confidence reports of each trial, in accordance with Garfinkel et al.^3^. High positive correlation scores indicated better IAwn, while low or negative scores reflected poorer IAw. The calculation was not possible for 8 patients due to constant confidence rating (e.g., 10, 10, 10) lacking variability, essential to compute correlation. Therefore we introduced the DI, an alternative outcome previously described^37^, by converting the confidence ratings (0–10) to percentages, and calculating an absolute difference between accuracy and confidence for each trial (| ‘Interoceptive accuracy’ – ‘confidence’|), followed by averaging across the trials. A DI near 0 indicates strong interoceptive metacognition (high concordance between accuracy and confidence), with deviations showing mismatched insight: negative DI scores suggest overconfidence with low performance, and positive scores indicate under confidence despite high performance in the heartbeat task.

#### Interoceptive sensibility

IS was measured using the Multidimensional Assessment of Interoceptive Awareness (MAIA)^8^, which assesses seven dimensions of interoceptive sensibility through 33 items rated on a 6-point Likert scale “0 (“never”) to 5 (“always”), where patients must judge the frequency that each statement applies in their daily lives. These dimensions are: (1) Noticing, the awareness of one’s body sensations (3 items); (2) Not-distracting, the tendency not to ignore or distract oneself from sensations of pain or discomfort (4 items); (3) Not-worrying, the tendency not to experience emotional distress or worry with sensations of pain or discomfort (4 items); (4) Attention regulation, the ability to sustain and control attention to body sensation (7 items); (5) Emotional awareness, the awareness of the connection between body sensations and emotional states (5 items); (6) Self-regulation, the ability to regulate psychological distress by attention to body sensations (7 items); (7) Trusting: the experience of one’s body as safe and trustworthy (3 items). The score per scale was calculated by averaging items belonging to the scale. The translated Portuguese version with good psychometric properties (α = 0.79 to 0.90 across subscales) was used^38^.

### Cognitive assessment

Executive functions involve higher-order cognitive processes and are highly modulated by emotional and motivational processes^39^. Previous studies demonstrated that fibromyalgia patients might have lower performance than non-clinical groups in these executive tasks, particularly those requiring working memory and inhibitory control^22,40–42^. Based on these studies, we assessed executive functions using two easy-to-administrate and well-validated tasks: Digit-span to measure working memory and Stroop task to measure inhibitory control.

#### Digit-span

The Digit-span task is part of the Wechsler memory scale^43^ and composed of two subtests: Digit-span forward and the Digit-span backwards. In Digit-span forward, participants are instructed to reproduce a sequence of numbers in the same order as it was presented by the assessor. Conversely, the Digit-span backwards requires participants to reproduce the presented sequence of numbers in reverse order. The difficulty of these tasks increases gradually, starting with two digits and progressing up to nine, with two attempts per level. Scoring was based on the total number of digits correctly recalled in each subtest. Three scores were derived from these tests, Digit-span forward and Digit-span backward, measuring short-term and working memory respectively and Digit-span total as a measure of general memory performance, summing total number of digits correctly recalled in both subtests. The Wechsler Memory Scale’s Portuguese version^43^ was used, presenting good internal consistency (α = 0.89).

#### Stroop

The classical Stroop test measures executive functioning, like conflict monitoring and inhibitory control^44^. This test consists of three tasks, each completed in 45 s. While in the first task, participants are requested to read aloud 100 colour names written on a sheet of paper, in the second task participants must name the colour in which the letters of the “XXXX” were written. In the third task, participants were presented with words of colours names (e.g., “GREEN”) printed in incongruent colours (printed in blue), requiring the participant to inhibit the automatic reading response (green) and instead identify the colour of the printed letters (blue). Four outcomes were derived from these tests, each score reflecting the number of stimuli correctly identified in Stroop word, Stroop colour, Stroop colour-word. The interference score was calculated by the intersection of the raw scores obtained in the first task (word) and the second task (colour), as specified in a table provided in the Stroop Manual – Test Colours and Words, adopting normative data from the Portuguese^45^. Based on this calculation method, higher interference scores indicate better inhibitory control. The Portuguese validation of this instrument demonstrates good psychometric properties (test–retest reliability coefficients between 0.78 and 0.9) and was used.

### Clinical assessment

Clinical characteristics and medication intake (Table S1) were assessed via self-reported questionnaires including the Brief Pain Inventory (BPI), Functional Assessment of Chronic Illness Therapy (FACIT), Fibromyalgia Impact Questionnaire (FIQ), Hospital Depression and Anxiety Scale (HADS). The description of these questionnaires is elaborated in the supplementary materials.

### Study design

The study protocol, approved by the Universidade Católica Portuguesa and the Hospital Ethical Committee (Comissão de Ética para a Saúde do Centro Hospitalar de Lisboa Ocidental) involved additional assessment of pain sensitivity and modulation of these patients. However these results will not be reported here.

**Fig. 1:**
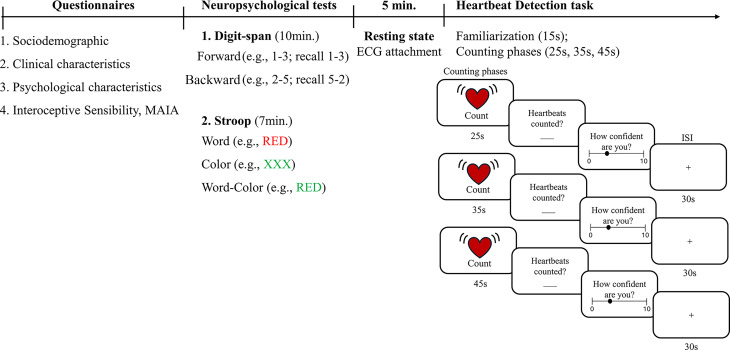
Experimental design. Participants first completed self-report questionnaires (including MAIA), followed by cognitive (Digit-span and Stroop tests) and interoceptive tasks (heartbeat detection task and confidence rating).

Patients diagnosed with Fibromyalgia at the Rheumatology Department were referred to participate, confirming diagnostic criteria. After signing informed consent, participants completed a battery of questionnaires assessing sociodemographic, clinical, and psychological characteristics, followed by the neuropsychological tests (Digit-span task and Stroop Test). After completion of these tasks, following a 5-min period of rest, the patients completed the HDT (Fig. 1). Participants were acknowledged for their contribution.

### Statistical analysis

Statistical analyses were conducted with the SPSS Statistical Package for the Social Sciences software (IBM SPSS Statistics, version 25.0, https://www.ibm.com/products/spss-statistics), and RStudio software (Version 2024.09.0 + 375 for Windows, https://posit.co), and RStudio software was used for visualization purposes. Descriptive statistics were used to explore sociodemographic, clinical characteristics and main outcomes distributions, followed by normality tests, and supplemented by skewness and kurtosis inspection.

Parametric (Pearson, r) and non-parametric (Spearman, rs) correlational tests were employed to explore associations among clinical characteristics, interoceptive outcomes and cognitive tasks. Correlation estimates (r, rs) are reported with 95% Confidence Intervals (95% CI). Correlational effect sizes were interpreted as small (r = 0.10), medium (r = 0.30) or large/strong (r = 0.50)^46^. An exploratory approach was adopted to further investigate the relationship between clinical characteristics, interoception dimensions, and performance in the cognitive tasks.

To assess the potential moderating role of the clinical characteristics on the prediction of the Digit-span backward by the IAc outcome, which evidenced a trend towards significant correlation, we used Hayes model 1 from the PROCESS macro for SPSS (version 4.2)^47^. We tested the prediction of the Digit-span backward task by the IAc at three levels of each moderator: low, mean, and high (± 1 standard deviation from the mean). Post hoc Johnson-Neyman analysis were employed to identify the significance regions of the moderator.

Data are presented as mean (standard deviation, SD), medians and interquartile ranges for descriptive variables (in tables) and statistical significance was set at α value of < 0.05 for all analyses.

## Results

### Patients’ sociodemographic characterization and medical information

Thirty female patients diagnosed with Fibromyalgia were enrolled, but due to personal restraints, one of the participants didn’t complete the study protocol and data of 29 participants was analysed. The mean age was 50.41 (SD 10.34; range:30–76). Amongst 51.7% of the participants had more than 9 years of education, and 65.5% were married. The median time since diagnosis was 3 years (IQR: 2–8), but symptoms had been present for a median of 10 years (IQR: 5–21), ranging from 2 to 46 years. Most participants were using pain medication (79.3%) to address their symptoms (supplementary material—Table S1). Detailed sociodemographic, clinical and psychological characteristics are depicted in (Table 1).

**Table 1:** Participants’ sociodemographic and clinical characteristics (N = 29).

	Mean (SD)	Median (IQR)	Range (Min.-Max.)
**Sociodemographic**			
Age	50.41 (10.34)	52.00 (41.50–57.00)	30–76
BMI	27.03 (4.33)	26.22 (23.34–29.27)	20.57–36.05
Education (years)	10.25 (4.22)	12.00 (6.00–14.25)	4–17
**Clinical characteristics** ^1^			
Years of symptoms	13.96 (11.21)	10.00 (5–21)	2–46
Years of diagnose	5.70 (4.96)	3.00 (2–8)	0–17
FIQ total	64.29 (17.32)	64.98 (53.68–78.11)	15.58–95.76
Physical impact	3.15 (2.11)	3.33 (1.06–4.49)	0–8.17
Feeling good	6.38 (2.78)	7.15 (4.29–8.94)	0–10.00
Work missed	3.18 (3.74)	1.43 (0–7.15)	0–12.87
Symptoms	50.82 (13.26)	50.50 (44.50 –61.00)	7.00–69.00
BPI			
Severity score	5.51 (1.99)	5.75 (4.62–5.88)	0–9
Interference score	5.81 (2.26)	6.14 (4.36–7.36)	0–9.43
HADS total	19.96 (7.10)	21.00 (16.25–25.00)	5–34
Anxiety	11.46 (3.86)	12.00 (8.25–14.75)	3–18
Depression	8.5 (3.88)	9.00 (6.25–10.75)	0–16
FACIT total	31.10 (12.19)	36.00 (22.00–39.00)	3–49

^1^*FIQ,* Fibromyalgia Impact Questionnaire, *BPI,* Brief Pain Inventory, *HADS,* Hospital Anxiety and Depression Scale, *FACIT, Fatigue* Functional Assessment of Chronic Illness Therapy‐Fatigue.

### Descriptives of the interoceptive assessment

Table 2 summarizes the descriptive statistics for the interoceptive measures, showing a wide range of scores and substantial variability among participants across measures.

**Table 2:** Descriptives of the different interoceptive outcomes organized by dimensions.

	Mean (SD)	Median (IQR)	Min. – Max
**Interoceptive accuracy** (n = 28)			
Accuracy	0.53 (0.30)	0.51 (0.33–0.79)	0–0.98
**Interoceptive sensibility** (n = 29)			
Noticing	4.08 (0.71)	4.33 (3.50–4.67)	2.67–5.00
Not-distracting	1.10 (0.93)	0.88 (0.31–1.69)	0–3.00
Not-worrying	2.54 (0.60)	2.50 (2.50–2.94)	0.75–3.50
Attention regulation	2.88 (1.11)	2.86 (2.15–3.71)	0–4.71
Emotional awareness	3.97 (0.93)	4.10 (3.60–4.60)	0.40–5.00
Self-regulation	2.70 (1.16)	2.86 (2.00–3.78)	0.43–5.00
Trusting	2.44 (1.48)	2.00 (1.17–3.67)	0–5.00
**Interoceptive awareness**			
Confidence Avg. (CA) (n = 29)	6.40 (2.60)	6.67 (4.67–8.33)	1.00–10.00
Correlation (n = 20)	0.23 (0.78)	0.59 (−0.68–0.97)	−0.96–1.00
Discrepancy index (DI) (n = 28)	0.37 (0.29)	0.28 (0.16–6.45)	0–1.00

### Descriptives of the neuropsychological assessment

Table 3 depicts the descriptive statistics of the Digit-span and Stroop tasks outcomes, with results aligning with normal Portuguese parameters for age and education, despite the wide score ranges.

**Table 3:** Descriptive statistics of the neuropsychological assessment.

	Mean (SD)	Median (IQR)	Range (min–max)
**Digit–span scores**			
Forward	8.14 (2.17)	8.00 (6.00–10.00)	4.00–12.00
Backward	5.24 (1.85)	6.00 (4.00–6.50)	2.00–8.00
Total	13.38 (3.31)	14.00 (10.50–16.00)	8.00–20.00
**Stroop task scores**			
Words	78.07 (14.59)	79.00 (65.00–88.00)	55.00–107.00
Colors	61.08 (11.37)	60.50 (48.00–68.25)	42.00–87.00
Color-word	32.31 (7.63)	29.50 (26.00–40.00)	20.00–47.00
Interference	−1.35 (5.01)	−0.50 (−5.00–3.00)	−11.00–7.00

### Associations between clinical characteristics and interoceptive outcomes

No correlations were found between sociodemographic or clinical characteristics (Table 1) and IAc (all p > 0.05). However, clinical symptoms, measured using the BPI, FACIT, HADS, and FIQ showed significant correlations with IS (as measured by the MAIA, (see supplementary materials)). Furthermore, the number of years since the fibromyalgia diagnosis was positively correlated with the MAIA subscales of self-regulation (r = 0.529, p = 0.011, 95%CI [0.138,0.777]) and trusting (r = 0.424, p = 0.049, 95% CI [0.003, 0.718]). In addition, the number of years since the symptom’s onset was positively correlated with IAw, as measured by the CA (rs = 0.444, p = 0.020, 95% CI [0.065, 0.711]).

### Associations between the clinical characteristics and neuropsychological assessment

The BPI severity score was negatively correlated with Stroop task interference score (r = −0.423, p = 0.032, 95% CI [−0.696, −0.042]).

### Associations between interoceptive accuracy and neuropsychological assessment

**Fig. 2:**
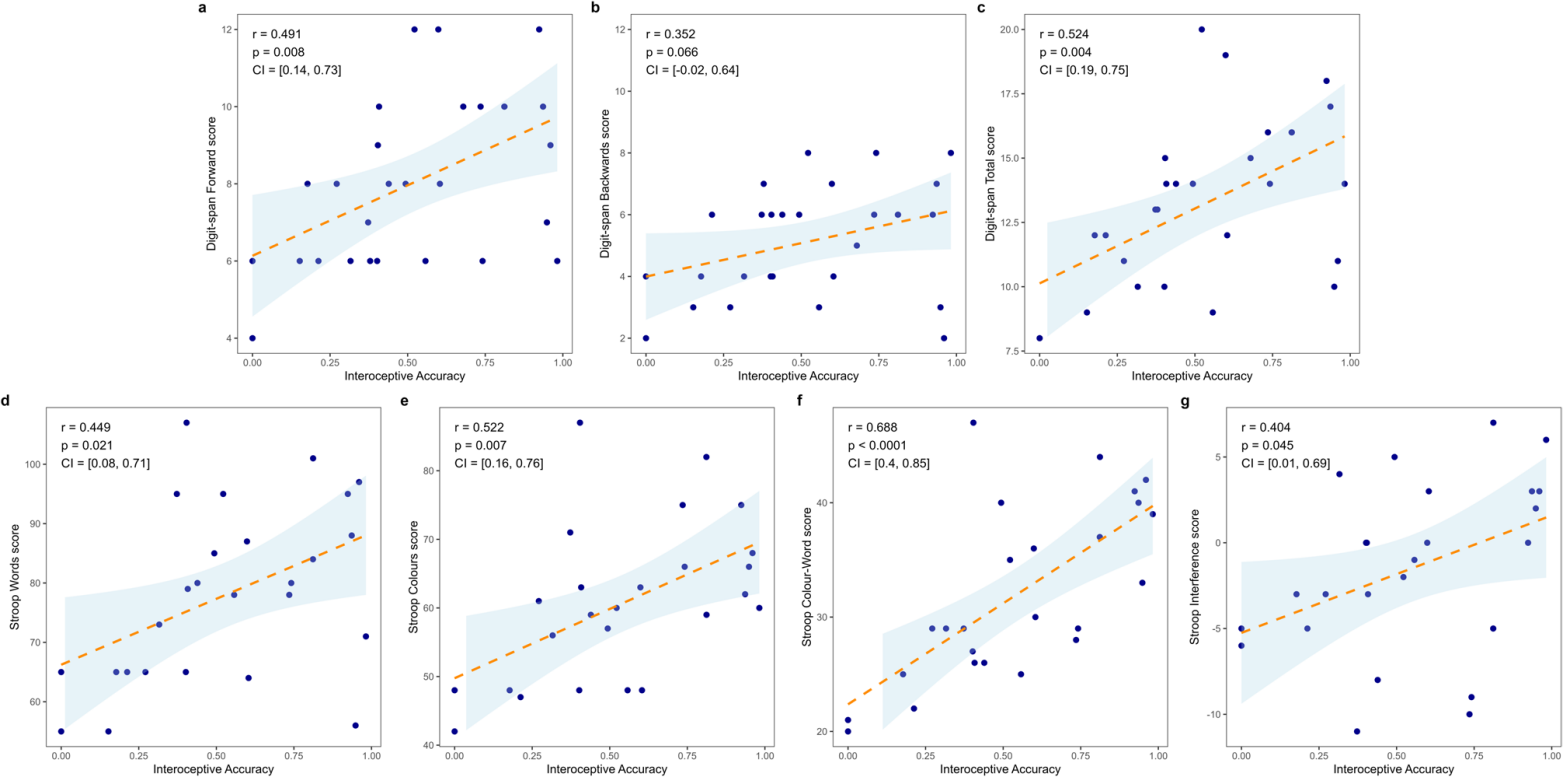
Associations between interoceptive accuracy and performance on Digit-span (**a**–**c**) and Stroop tasks (**d**–**g**).

Figure 2 illustrate the Pearson correlations found for IAc and cognitive tasks performance. IAc was positively correlated with Digit-span forward (r = 0.491, p = 0.008, 95%CI [0.144, 0.730]) and Digit-span total (r = 0.524, p = 0.004, 95% CI [0.188, 0.750]), both showing moderate to large effect sizes, respectively. A trend towards significance was also observed for the Digit-span backward task (r = 0.352, p = 0.066). Additionally, IAc showed strong positive correlation with the Stroop task scores: in word (r = 0.449, p = 0.021, 95% CI [0.075, 0.713]), colour (r = 0.522, p = 0.007, 95% CI [0.159, 0.760]), colour-word (r = 0.688, p < 0.001, 95% CI [0.403, 0.852]) and interference (r = 0.404, p = 0.045, 95% CI [0.011, 0.689]), showing medium to large effect sizes.

### Interoceptive sensibility and neuropsychological assessment

**Fig. 3:**
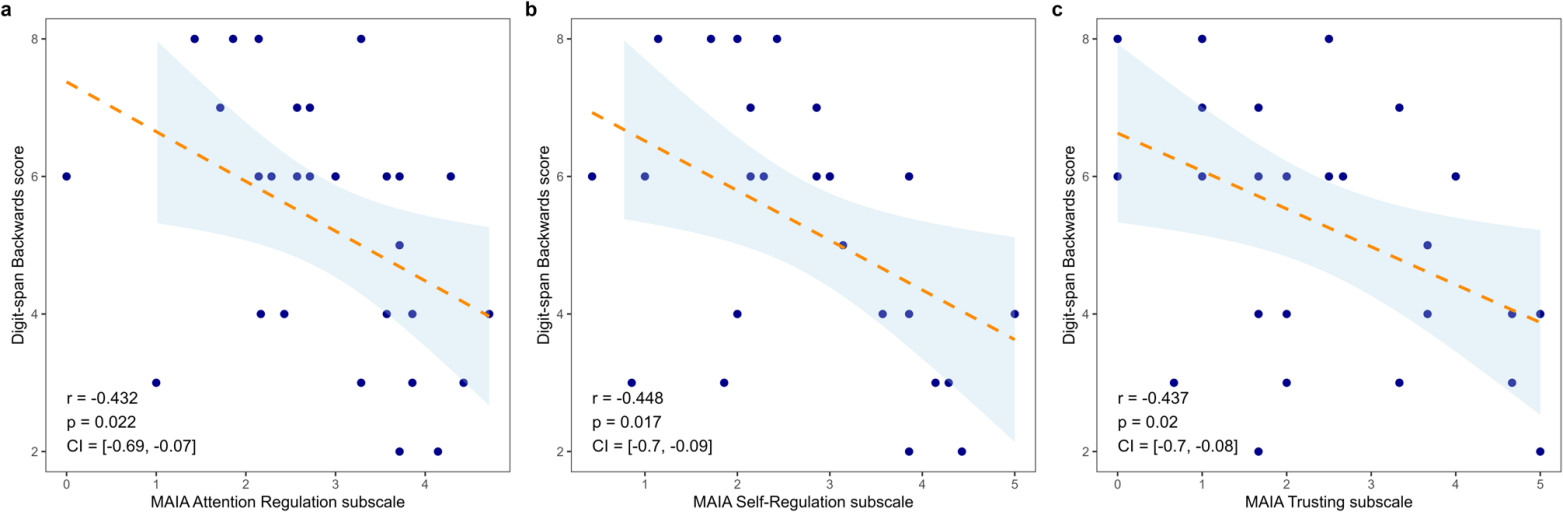
Associations between interoceptive sensibility and performance on Digit-span task. A negative correlation was found between (**a**) Attention regulation, (**b**) Self-regulation and (**c**) Trusting with the Digit-span backward task.

In the IS dimension, the MAIA subscales of attention regulation (r = −0.432, p = 0.022, 95% CI [−0.693, −0.070]), self-regulation (r = −0.448, p = 0.017, 95% CI [−0.704, −0.090]) and trusting (r = −0.437, p = 0.020, 95% CI [−0.697, −0.077]) showed moderate negative correlations with the Digit-span backward task (Fig. 3). No other significant correlations were observed between IS and cognitive tasks.

### Interoceptive awareness and neuropsychological assessment

Significant negative correlations were found between CA and DI and performance on the Digit-span forward (CA: r = −0.480, p = 0.008, 95% CI [−0.716, −0.129]; DI: r = −0.402, p = 0.034, 95% CI [−0.674, −0.034]) and as well as on the Digit-span total scores (CA: r = −0.484, p = 0.008, 95% CI [−0.718, −0.134]; DI: r = −0.528, p = 0.004, 95% CI [−0.753, −0.193]). Significant negative correlations were also observed between CA and DI with the Stroop task performance: Stroop word (CA: r = −0.582, p = 0.001 95% CI [−0.783, −0.249]; DI: r = −0.493, p = 0.011, 95% CI [−0.739, −0.130]), Stroop colour (DI: r = −0.485, p = 0.014, 95% CI [−0.739, −0.111]) and Stroop colour-word (CA: r = −0.410, p = 0.038, 95% CI [−0.683, −0.018]). All significant correlations showed moderate to large effect sizes.

### Clinical characteristics and interoceptive accuracy as a predictor of working memory

Considering a trend towards significance found for the correlation between IAc and Digit-span backward, moderation analyses were performed to explore if clinical characteristics could potentially moderate this relationship. The model with FIQ symptoms scale and IAc was significant (F(3, 23) = 3.800, p = 0.024), and explained 33% of the variance in Digit-span Backward score. The FIQ symptoms scale showed a significant interaction with IAc in predicting the Digit-span backward (B = 0.183, SE = 0.087, p = 0.045). Simple slopes analysis revealed that the IAc significantly predicted Digit-span backward for individuals with average (B = 2.175, SE = 1.03, p = 0.046, 95% CI [0.038, 4.311]) and higher symptom levels (B = 4.644, SE = 1.48, p = 0.004, 95% CI [1.579, 7.709]), but not for those with low symptom levels (B = -0.294, p = 0.858). The interaction explained an additional 13% of the variance of the Digit-span backward, ΔR^2^ = 0.130, for values above 50.476, with 48.15% of the participants scoring above this threshold and suggesting that as symptom severity increases, the ability to accurately sense and respond to bodily signals might become more critical for maintaining cognitive functions like working memory.

## Discussion

The current study investigated the relationship between interoceptive dimensions and cognitive functioning in fibromyalgia patients. We found that two domains of interoception relate to cognitive performance differently: a lower IAc was associated with lower cognitive performance, notably in working memory and inhibitory control tasks, and higher IS on attention regulation, self-regulation, and trust in body states was associated with lower performance in the working memory task.

A growing body of literature has previously demonstrated that interoception is associated with emotional well-being and performance in emotional and self-regulatory tasks both in healthy and clinical groups^19,48–52^. In the cognitive domain, most of the data highlights the associations between better IAc and IS and memory functioning, particularly in hippocampal-dependent learning and memory performance measured by neuropsychological tests in healthy individuals^53–58^. Additionally, the relationship between interoception and executive functioning was explored by Werner et al.^52^, which demonstrated that individuals with higher scores on the heartbeat detection task chose significantly less disadvantageous and more advantageous options in the Iowa Gambling Task compared to participants with low interoceptive scores. Other studies on non-clinical groups found that inducing changes in the perception of body states after vaccination resulted in changes in the brain areas recruited when the individuals performed the Stroop task^30^ and that individuals with high interoception could have better prospective memory^31^.

Our study expands these findings to a clinical group, suggesting that an enhanced ability to perceive bodily states is related to the ability to respond to tasks requiring working memory and inhibitory control in a clinical population vulnerable to cognitive dysfunction.

The specific mechanisms underlying this relation are yet to be defined. However, better heartbeat detection may contribute to error detection and cognitive resource allocation during demanding tasks like the Stroop task. This task requires the ability to inhibit automatic responses and rapidly overcome errors. The body signals induced by errors are essential for stimulating changes in performance and may drive reappraisal strategies and induce behavioral changes (for a review see Di Gregorio et al.^59^). This feedback mechanism involves self-regulation and is associated with the activity of the anterior cingulate cortex, an essential element of the interoceptive brain network and cognitive-emotional interface^60–63^. Thus, we hypothesized that individuals with higher accuracy in detecting heartbeats may have perceived the errors easily and quickly directed their cognitive resources to overcome them during the tasks. In addition, our results emphasize the interaction of emotional and cognitive domains in the performance of executive functioning^39,61,62^ and its associations with interoception abilities to fully understand the cognitive of chronic pain patients. Further studies assessing the role of attention (e.g., selective attention abilities) and using more precise and sophisticated psychophysical methods may allow a direct assessment of this hypothesis.

Interestingly, on the other hand, we found evidence that in fibromyalgia patients higher IS and IAw may have deleterious effects on cognitive functioning, eventually exhausting resources that could have been directed toward the cognitive tasks. In line with previous studies and a recent systematic review and meta-analyses in chronic pain patients^16,63,64^, we found that the higher the number of years of symptoms of fibromyalgia, the higher the self-reported ability to trust in body signals and to self-regulate them, and the higher the IAw. This increase in the scores of interoceptive dimensions may indicate the patients’ need to consciously and daily integrate abnormal body states due to chronic pain and other symptoms. The resources for this constant monitoring may exhaust the patient’s cognitive resources. The negative correlations found between ISn and Digit-span scores suggest that a higher tendency to direct cognitive resources toward detecting and monitoring internal states (for example, the painful) may be related to decreased ability to manipulate complex cognitive tasks, particularly if the patients have stronger symptomatology. Compared to other domains of IS, self-attention, self-regulation, and trust are processes that involve more than perceiving the bodily signals; they involve a higher order process on these signals that may require higher cognitive resources. If the individual allocates increased resources to regulate the body state, it may deplete the ability to allocate cognitive resources to other external, non-body cognitive tasks^65^. Indeed, the findings of lower cognitive abilities in chronic pain patients have been interpreted as a consequence of the limited resources of the individuals and the competition that processing painful and cognitive stimuli implies on brain networks^66^. Further studies measuring attentional abilities in these patients may offer valuable insights and support to this hypothesis^39^. Other plausible explanations for these results could involve alternative neurocognitive mechanisms, which include hypervigilance and catastrophizing^21,25^ or may be a consequence of the patient’s difficulty in providing a reliable view of their interoceptive sensibility abilities^4^. To enhance the clarity of these results, incorporating a control group could provide information concerning the specificity of these results, demonstrating if and to what extent these relations between interoception dimensions and cognitive abilities are a general phenomenon or a specific observation in this clinical group.

In summary, fibromyalgia patients with a lower ability to accurately perceive bodily signals, particularly on the cardiac modality, may have difficulties using these key signals to direct cognitive abilities. The detection of bodily signals, specifically cardiac signals, eventually related to error detection, regulation, and emotional state, are predictors of cognitive performance in fibromyalgia patients. However, paradoxically, efforts to self-regulate symptoms and body states may contribute to difficulty manipulating cognitive information.

These findings deserve further investigation to overcome their limitations, such as (1) the absence of control of attentional abilities, psychological symptoms and medication use, (2) the correlational nature, (3) the single-site recruitment, and (4) the small sample size at the lower threshold for statistical power, which may not fully represent our population of interest, limit the generalizability of the findings of the current study, and increase the risk of statistical error. In addition, (5) heartbeat detection tasks, particularly when using fixed order estimation intervals, raises reliability and validity concerns, as it can be influenced by physiological and psychological factors, including learning and beliefs about one’s heartbeat and time estimation. Therefore, the results should be considered hypothesis-generating and require confirmation in future studies.

Nonetheless, the current study highlights the urge to disentangle the role of different interoceptive domains on cognitive performance and to use this knowledge to provide new avenues for specific therapeutic targets on each interoception domain and on which interventions (e.g., interoceptive vs. cognitive training) could minimize cognitive dysfunction and lower the suffering experience of fibromyalgia patients. The results of this study underscore the relevance of including assessment and intervention about the perception of inner states^67^, eventually by helping patients to be more precise and increase their knowledge about body perceptions while avoiding an excessive focus on these sensations, which could involve over-attending and having deleterious consequences in the ability to respond to outside demands. Additional studies may help determine the key interoception dimensions that should be subjected to intervention and allow a more detailed view of the critical underlying mechanisms for further clinical improvement.

## Supplementary Information

Supplementary Information.

## Data Availability

Data is available upon reasonable request from RC.
