# Supplementary material for: Disentangling interoception and its links to cognitive functioning in fibromyalgia
Source: Sci Rep. 2025 Aug 26;15:31403. doi: 10.1038/s41598-025-15087-5 (PMC12381188; doi:10.1038/s41598-025-15087-5)
Supplement: Supplementary file 1 — Supplementary Information. [file 41598_2025_15087_MOESM1_ESM.docx]

**Supplementary Materials**

**Methods**

**1. Questionnaire’s description**

**1.1. Brief Pain Inventory (BPI)**

The BPI is a pain self-report measure assessing pain in a multidimensional perspective [1]. It includes 15 items revolving on the existence of pain, its severity, location, therapeutics, and functional impact. The Portuguese version of the BPI, which revealed good psychometric properties (α = 0.89) was used [2].

**1.2. Fibromyalgia Impact Questionnaire (FIQ)**

The FIQ is used to assess health problems related to FM and its impact on daily living [3]. It comprises information about function, overall impact, and symptoms. The FIQ Physical Functioning domain is based on the patient answers to 11 items rated on a 4-point scale ranging from "Always" to "Never". The Portuguese version, developed by Rosado et al. [4] that has demonstrated good psychometric properties (α = 0.83) was used.

**1.3. Hospital Depression and Anxiety Scale (HADS)**

The Hospital Depression and Anxiety Scale is a brief instrument commonly used to assess anxiety and depression in physically ill populations [5]. It has 14 items and two subscales: Depression (7 items) and Anxiety (7 items). The validated Portuguese version of this instrument showed good internal consistency (α = 0.76 for anxiety and α = 0.81 for depression) was used [6].

**1.4. FACIT**

Fatigue is a symptom present in patients with chronic diseases. The FACIT fatigue test isself-reported, demonstrates excellent internal consistency (α = 0.93) and measures the individual's perception and severity of fatigue and its impact on daily activities. The FACIT Fatigue scale (version 4) consists of 13 items and each item is averaged on a 4-point Likert scale [7]. Total improvement ranges from 0 to 52. Less fatigue is demonstrated by achieving a high score on the scale.

**Results**

**2.1. Medication intake**

**Table S1. Descriptives of medication intake.**

| Medication^1^ | N (%) |
| --- | --- |
| Analgesic  Psychotropics  Rheumatic  Hormonal | 23 (79.3%)  15 (51.7%)  3 (10.3%)  3 (10.3%) |

Note^1^. Medications: analgesic (non-steroidal anti-inflammatory, analgesic, weak opioids), psychotropics (anticonvulsants, antidepressants, anxiolytics, antipsychotics, amphetamines), rheumatic (anti-rheumatic, biological, corticosteroids), hormonal (thyroid-related, oral contraceptives, menopause-related).

**Associations between Interoceptive Sensibility and Clinical characteristics**

BPI interference correlated negatively with Trusting subscale (r = -0.501, p = 0.007 95%CI [-0.736, -0.157].

HADS anxiety, depression and total correlated with Noticing (Anxiety: r = -0.425, p = 0.024, 95%CI [-0.689, -0.062]; Depression: r = -0.433, p = 0.021, 95%CI [-0.694,-0.071]; Total: r = -0.468, p = 0.012, 95%CI [-0.716, -0.115]), Not-worrying (Anxiety: rs = -0.482, p=0.011, 95%CI [-0.734, -0.113]; Total: rs = -0.474, p = 0.013, 95%CI [-0.729, -0.103]), Attention regulation (Anxiety: r = -0.434, p = 0.024, 95%CI [-0.699, -0.064]; Total: r = -0.394, p = 0.042, 95%CI [-0.674, -0.017]) and Self-regulation (Anxiety: r = -0.469, p=0.014, 95%CI [-0.721, -0.108]; Depression; r=-0.469, p=0.014) and Trusting (Anxiety: r=-0.579, p=0.002, Depression: r = -0.380, p = 0.050, 95%CI [-0.664, 0.000]; Total: r = -0.462, p = 0.015, 95%CI [-0.716, -0.100]).

FIQ questionnaire, namely the physical impact, feel good, missed work days, symptoms subscales and Total correlated with Not Distracting (Phys. Impact: rs = 0.729, p < 0.001, 95%CI [0.474, 0.871]; FIQ Total: rs=0.396, p=0.050, 95%CI [-0.011, 0.691]), Attentional regulation (Phys. Impact: r = -0.585, p = 0.001, 95%CI [-0.790, -0.264]; Symptoms: r = -0.406, p = 0.036, 95%CI [-0.681, -0.031]; FIQ Total: r = -0.490, p = 0.013, 95%CI [-0.742, -0.118]), Self-regulation (Phys. Impact: r = -0.435, p = 0.023, 95%CI [-0.699, -0.065]; Symptoms: r = -0.487, p = 0.010, 95%CI [-0.732, -0.132]; FIQ Total: r = -0.503, p = 0.010), 95%CI [-0.749, -0.134], and Trusting (Phys. Impact: r = -0.643, p < 0.001, 95%CI[-0.822, -0.348]; Feel Good: r = -0.496, p = 0.012, 95%CI [-0.745, -0.126]; Missed work: rs = -0.390, p = 0.049, [-0.682, 0.009]; Symptoms: r = -0.469, p = 0.014, 95%CI [-0.720, -0.108]; FIQ Total: r = -0.643, p < 0.001, 95%CI [-0.828, -0.332]).

FACIT correlated negatively with Attention regulation (r = -0.389, p = 0.041, 95%CI [-0.666, -0.019]) and Trusting (r = -0.527, p = 0.004, 95%CI [-0.752, -0.192]) subscales.

**Associations between Interoceptive Accuracy and neuropsychological assessment while controlling for age and education as covariates**

The IAc was positively correlated with Digit-span Total (r = 0.405, p = 0.045, 95%CI [-0.021, 0.201]) and a trend towards significance with the Digit-span Forward (r = 0.362, p = 0.075, 95%CI [-0.026, 0.207]) and no association with Digit-span Backwards task (r = 0.248, p = 0.232). The IAc was also positively correlated with the Stroop task Colour (r = 0.469, p = 0.027, 95%CI [-0.009, 0.786]), Colour-Word (r = 0.653, p < 0.001, 95%CI [0.261, 0.865]), a trend towards significance for the Interference (r = 0.379, p = 0.082) and no associations with Word task (r = 0.311, p = 0.148).

**Interoceptive Sensibility and neuropsychological assessment while controlling for age and education as covariates**

In the IS dimension, the MAIA subscales of Attention regulation (r = -0.415, p = 0.039, 95%CI [-0.749, -0.044]), Self-regulation (r = -0.485, p = 0.014, 95%CI [-0.753, -0.083]) and Trusting (r = -0.473, p = 0.017, 95%CI [-0.765, -0.122]) showed negative correlations with the Digit-span Backwards Task (figure 2). No other correlations between IS and cognitive tasks were found.

**Interoceptive Awareness and neuropsychological assessment** **while controlling for age and education as covariates**

Negative trends were found between CA with the Digit-span Forward (r = -0.343, p=0.086) and Digit-span Total scores (r = -0.346, p = 0.084). Significant correlations between CA and DI with the Stroop task were also found: Stroop Word (CA: r = -0.505, p = 0.012 95%CI[-0.766, -0.151]), Stroop Colour (DI: r = -0.451, p = 0.035, 95%CI[-0.765, -0.042]).

**References**

1. Cleeland C. S. & Ryan K. M. Pain assessment: global use of the Brief Pain Inventory. *Ann Acad Med Singap* **23**, 129–138 (1994).
2. Azevedo, L., et al. Tradução, adaptação cultural e estudo multicêntrico de validação de instrumentos para rastreio e avaliação do impacto da dor crónica (Translation, cultural adaptation and multicentric validation study of chronic pain screening and impact assessment instruments). *Dor* **15**, 6-56 (2007).
3. Burckhardt, C., Clark, S., & Bennett, R. The Fibromyalgia Impact Questionnaire: Development and validation. *J Rheumatol* **18**, 728–734 (1991).
4. Rosado, M., Pereira, J., Fonseca, J., & Branco, J. Adaptação cultural e validação do “Fibromyalgia Impact Questionnaire” – versão portuguesa. *Acta Reuma Port* **31**, 157–165 (2006)
5. Zigmond, A., & Snaith, R. The Hospital Anxiety and Depression Scale. *Acta Psychiatrica Scandinavica* **67(6)**, 361-370 (1983). doi: 10.1111/j.1600-0447.1983.tb09716.x
6. McIntyre, M. T., Pereira, G., Soares, V., Gouveis, J., & Silva, S. Escala de Ansiedade e Depressão Hospitalar. Versão Portuguesa de investigação. *Universidade do Minho, Departamento de Psicologia* (1999).
7. Acaster S, Dickerhoof R, DeBusk K, Bernard K, Strauss W, Allen LF. Qualitative and quantitative validation of the FACIT-fatigue scale in iron deficiency anemia. Health Qual Life Outcomes. **17(13)**, 60. (2015).
